# Diffusion kurtosis imaging and diffusion tensor imaging parameters applied to white matter and gray matter of patients with anti-N-methyl-D-aspartate receptor encephalitis

**DOI:** 10.3389/fnins.2022.1030230

**Published:** 2022-11-24

**Authors:** Hanjing Liu, Yayun Xiang, Junhang Liu, Jinzhou Feng, Silin Du, Tianyou Luo, Yongmei Li, Chun Zeng

**Affiliations:** ^1^Department of Radiology, The First Affiliated Hospital of Chongqing Medical University, Chongqing, China; ^2^Department of Neurology, The First Affiliated Hospital of Chongqing Medical University, Chongqing, China

**Keywords:** anti-N-methyl-D-aspartate receptor encephalitis, microstructural changes, white matter, gray matter, diffusion kurtosis imaging, diffusion tensor imaging

## Abstract

**Objectives:**

To compare parameters of diffusion tensor imaging (DTI) and diffusion kurtosis imaging (DKI) to evaluate which can better describe the microstructural changes of anti-N-methyl-D-aspartate receptor (NMDAR) encephalitis patients and to characterize the non-Gaussian diffusion patterns of the whole brain and their correlation with neuropsychological impairments in these patients.

**Materials and methods:**

DTI and DKI parameters were measured in 57 patients with anti-NMDAR encephalitis and 42 healthy controls. Voxel-based analysis was used to evaluate group differences between white matter and gray matter separately. The modified Rankin Scale (mRS) was used to evaluate the severity of the neurofunctional recovery of patients, the Montreal Cognitive Assessment (MoCA) was used to assess global cognitive performance, and the Hamilton Depression Scale (HAMD) and fatigue severity scale (FSS) were used to evaluate depressive and fatigue states.

**Results:**

Patients with anti-NMDAR encephalitis showed significantly decreased radial kurtosis (RK) in the right extranucleus in white matter (*P* < 0.001) and notably decreased kurtosis fractional anisotropy (KFA) in the right precuneus, the right superior parietal gyrus (SPG), the left precuneus, left middle occipital gyrus, and left superior occipital gyrus in gray matter (*P* < 0.001). Gray matter regions with decreased KFA overlapped with those with decreased RK in the left middle temporal gyrus, superior temporal gyrus (STG), supramarginal gyrus (SMG), postcentral gyrus (POCG), inferior parietal but supramarginal gyrus, angular gyrus (IPL) and angular gyrus (ANG) (*P* < 0.001). The KFA and RK in the left ANG, IPL and POCG correlated positively with MoCA scores. KFA and RK in the left ANG, IPL, POCG and SMG correlated negatively with mRS scores. KFA in the left precuneus and right SPG as well as RK in the left STG correlated negatively with mRS scores. No significant correlation between KFA and RK in the abnormal brain regions and HAMD and FSS scores was found.

**Conclusion:**

The microstructural changes in gray matter were much more extensive than those in white matter in patients with anti-NMDAR encephalitis. The brain damage reflected by DKI parameters, which have higher sensitivity than parameters of DTI, correlated with cognitive impairment and the severity of the neurofunctional recovery.

## Introduction

Anti-N-methyl-D-aspartate receptor (NMDAR) encephalitis is the most common type of autoimmune encephalitis, with an incidence of 0.85 per million in children, while no data is available in adults (Guan et al., 2016). However, a previous study has shown that the global incidence of this disease has increased significantly, surpassing that of viral encephalitis in young people (Gable et al., 2012). Its clinical manifestations are complex and varied, often with abnormal mental behaviors, seizures, dysphasia, memory loss, involuntary movement, and autonomic dysfunction (Dalmau et al., 2019). Approximately 25% of patients may have severe disability or even die, with a mortality rate of 10%, whereas approximately 75% of patients experience sequelae, such as cognitive impairment (McKeon et al., 2018; Zhong et al., 2022). These consequences might have serious effects on patient prognosis.

Despite the severity and rapid progression of the clinical presentation, nearly half of the patients exhibited normal or non-specific manifestations on conventional magnetic resonance imaging (MRI) (Zhang et al., 2018). However, previous studies have confirmed that patients with anti-NMDAR encephalitis have alterations in the integrity and microstructural arrangement of brain white matter tissues, which can be detected by diffusion MRI techniques. Among these critical studies, previous reports have reached different conclusions by applying diffusion tensor imaging (DTI). One study reported that patients with anti-NMDAR encephalitis had extensive white matter changes (Finke et al., 2013), while another discovered that anti-NMDAR encephalitis patients had superficial white matter damage predominantly in the frontal and temporal lobes (Phillips et al., 2018). Consequently, in order to reach an objective conclusion, more sophisticated techniques must be used to investigate the plausible changes in white matter microstructure and associated pathophysiological pathways in patients with anti-NMDAR encephalitis.

Based on the assumption that the diffusion of water molecules obeys a Gaussian distribution, DTI is the most widely used technique to assess white matter changes. However, due to the presence of cell membranes, neurons, and other organelles in tissues, the diffusion of water molecules obeys a non-Gaussian distribution (Shi et al., 2021); hence, the parameters of DTI, including mean diffusivity (MD), axial diffusivity (AD), radial diffusivity (RD) and fractional anisotropy (FA), cannot describe water diffusion accurately. Diffusion kurtosis imaging (DKI) is regarded as a complementary technique for DTI, which can not only probe white matter changes accurately but can also assess the microstructural alternations in the gray matter which by the aggregation of many neuron cells and dendrites (Jensen et al., 2005). Studies have shown that the parameters of DKI, including mean kurtosis (MK), axial kurtosis (AK), radial kurtosis (RK) and kurtosis fractional anisotropy (KFA) is more suitable for assessing microstructural changes of white matter and gray matter regions with complex fiber arrangements (Qiao et al., 2020; Thaler et al., 2021).

In addition, voxel-based analysis (VBA) and region of interest (ROI)-wise analysis were applied in this study. VBA, a popular technique for assessing whole-brain DTI and/or DKI measurements, is an image postprocessing analysis method based on the evaluation of voxels. This method can avoid previous assumptions and automatically analyze the differences in various diffusion parameters in the whole brain including gray matter (Yuan et al., 2016; Yang et al., 2021; Wang et al., 2022). In this study, an ROI-wise VBA algorithm was used to detect brain regions with different diffusion parameters, and the diffusion parameters of corresponding brain regions were extracted and correlated with various clinical scales. This method is conducive to describing a comprehensive overview of anti-NMDAR patients’ brain microstructural damage. To our knowledge, the application of both DTI and DKI parameters to explore microstructure alternations in the whole brain, including white matter, gray matter and deep gray matter structures, in patients with anti-NMDAR encephalitis is rarely been reported.

Therefore, in this study, we aimed to characterize the non-Gaussian diffusion patterns of the whole brain in patients with anti-NMDAR encephalitis using DKI. The DKI parameters including MK, AK, RK and KFA and DKI-derived DTI parameters including MD, AD, RD and FA were used to detect brain microstructural changes. We compared the two methods to evaluate which method can better describe the microstructural changes of anti-NMDAR encephalitis patients. Our hypothesis was that DKI parameters might be more helpful in detecting brain microstructural changes in patients with anti-NMDAR encephalitis after the acute stage than DTI parameters, and these changes were related to patients’ neuropsychological impairments.

## Materials and methods

### Participants

Fifty-seven patients with anti-NMDAR encephalitis after the acute stage were recruited from the First Affiliated Hospital of Chongqing Medical University, and 42 age-, sex- and education level matched healthy controls (HCs) were recruited from the community. All the participants were enrolled between October 2015 and April 2022. All patients met the diagnostic criteria for anti-NMDAR encephalitis recommended by Lancet Neuro in 2016 (Graus et al., 2016) which included (1) acute onset of one or more of the following eight major clinical manifestations: psychosis, memory impairment, speech impairment, seizures, movement disorder, disturbance of consciousness, autonomic dysfunction, and central hypoventilation; (2) positive immunoglobulin G (IgG) NMDAR antibodies in the serum and/or cerebrospinal fluid (CSF); and (3) reasonable exclusion of other disorders. Exclusion criteria for all patients included: (1) being in the acute stage; (2) having a history of other non-anti-NMDAR encephalitis psychoneurosis, craniocerebral trauma and surgery; (3) having MRI contraindications; and (4) having images with artifacts or poor quality. HCs were defined as those without a history of neurological or psychiatric disorders or any other organic disease related to the central nervous system. All participants were right-handed.

Two experienced neurologists independently assessed patients’ disease severity and neuropsychological performances at the time of the study based on a comprehensive set of scales. The modified Rankin Scale (mRS) was used to evaluate the severity of the neurofunctional recovery of patients. The Montreal Cognitive Assessment (MoCA) was used to assess the global cognitive performance of all participants. The Hamilton Depression Scale (HAMD) was used to evaluate the depressive state of participants. The fatigue severity scale (FSS) was used to evaluate fatigue and the impact of fatigue on daily life.

### Magnetic resonance imaging data acquisitions

All MR images were acquired on a 3.0T MR scanner (Magnetom Skyra, Siemens Healthcare GmbH, Erlangen, Germany) with a 32-channel head coil. DKI images with 3 b-values (*b* = 0, 1000 and 2000 s/mm^2^) along 30 diffusion directions were acquired by using a single-shot, spin-echo echo planar imaging (EPI) sequence. Other MR scanning parameters included: slices = 50, voxel = 2 × 2 × 2 mm^3^, field of view (FOV) = 250 mm^2^, repetition time (TR) = 4500 ms, echo time (TE) = 94 ms, acquisition time (TA) = 5:25 min, integrated parallel acquisition techniques (iPAT) acceleration factor = 4 (GRAPPA), and partial-Fourier = 6/8. A magnetization prepared rapid gradient-echo (MPRAGE) sequence was used to acquire anatomical T1-weighted images (T_1_WI). The parameters included: voxel = 1 × 1 × 1 mm^3^, FOV = 256 mm^2^, TR = 2300 ms, TE = 2.26 ms, slices = 192, inversion time = 900 ms, and flip angle = 8 deg.

### Data processing and voxel-based analysis

First, we checked the basic parameters of the images, including resolution, dimension information and so on, then we visually inspected the images to assess the quality of the images and removed the images with obvious head movements and artifacts. Second, all DICOM images were converted into Neuroimaging Informatics Technology Initiative (NIfTI) format using the MRIcron tool named dcm2nii^1^ (SedDB, RRID, SCR_002403). Third, converted data were then loaded into the “Eddy-current” toolbox of the Functional Magnetic Resonance Imaging of the Brain software library (FSL)^2^ (SedDB, RRID, SCR_00283) to perform motion and eddy currents distortion correction (Andersson et al., 2003). Forth, the kurtosis and diffusion tensors were estimated using a constrained linear least square (CLLS) algorithm model in Diffusional Kurtosis Estimator^3^ (Tabesh et al., 2011). DKI tensor fitting was performed with constrained linear weighted fitting and DTI tensor fitting was performed with linear weighted fitting to obtain the DKI- and DTI-based parametric maps, respectively. Fifth, all the parametric maps were then normalized to the standard Montreal Neurological Institute (MNI) space. To normalize all the b0 images into the T2W template of the statistical parametric mapping (SPM)^4^ (SedDB, RRID, SCR_007110), we applied a non-linear registration tool in SPM8 with a resolution of 2 × 2 × 2 mm^3^. Then, a full width at half maximum (FWHM) kernel of 6 × 6 × 6 mm^3^ was used to make these images averaged and smoothed to generate a new b0 template (Good et al., 2001). For each parametric map, voxel-wise *t*-test were conducted using the white matter, gray matter and deep gray matter masks with sex, age and education adjusted in SPM8. The MNI T1 template was segmented in FSL to generate the white matter and gray matter masks.

### Statistical analyses

Demographic information and clinical assessment were compared between anti-NMDAR patients and HCs using the chi-square test and Mann-Whitney *U* test. Data that followed a normal distribution are represented as the mean ± standard deviation, and data that did not follow a normal distribution are represented as the median and interquartile range. A two-sample *t*-test in SPM8 with age, sex and education as nuisance variables was performed to compare differences between groups in white matter, gray matter and deep gray matter, statistical results were presented using the xjView toolbox. The significance level was set at a cluster level of uncorrected *P* < 0.001. Cluster size > 30 contiguous voxels was considered statistically significant. To account for multiple comparisons across the whole brain, false discovery rate (FDR) correction was performed and *P* < 0.05 were considered to show a significant difference. The diffusion parameters of brain regions that showed significant differences in VBA were extracted. Then, Spearman correlational analysis was performed to explore the relationship between the diffusion parameters and clinical scales, including MoCA, HAMD, and FSS scales; Kendall correlation analysis was performed to explore the relationship between the diffusion parameters and mRS scores. The statistical analysis was performed with SPSS software version 23.0. The criterion for statistical significance was set at *P* value < 0.05.

## Results

### Demographic information and clinical scale scores

The demographics information and clinical scales of the participants are shown in Table 1. Fifty-seven anti-NMDAR patients (27 males, 30 females) and 42 HCs (16 males, 26 females) were included in the study. There was no statistically significant difference in age (*P* = 0.673), sex (*P* = 0.415) or education level (*P* = 0.139) between patients with anti-NMDAR encephalitis and HCs. The patients underwent clinical scale evaluation and MRI scanning 15.44 ± 9.08 months after clinical onset. Two patients were moderately disabled (mRS = 3), six patients fully recovered (mRS = 0), and 48 patients still had mild symptoms but were able to accomplish their daily routine (mRS = 1–2). Compared to HCs, the MoCA scores were significantly decreased (*P* < 0.001), while HAMD (*P* < 0.001) and FSS (*P* < 0.001) scores were increased significantly in patients with anti-NMDAR encephalitis.

**TABLE 1:** Demographics information and clinical scales of the participants.

	Patients (*n* = 57)	HCs (*n* = 42)	*P*-value
Age (years)	31 (23,46)	30 (25,46.5)	0.673*
Sex, male/female	27/30	16/26	0.415^#^
Education (years)	12 (9,12.5)	12 (9,18)	0.139*
mRS	1(1,2)	N/A	N/A
MoCA	23 (20,25)	28 (26,29)	*P* < 0.001*
HAMD	10 (6,16)	3 (1,7)	*P* < 0.001*
FSS	21 (13, 31)	13.33 ± 4.83	*P* < 0.001*

HCs, healthy controls; mRS, modified Rankin Scale; N/A, not applicable; MoCA, Montreal Cognitive Assessment; HAMD, Hamilton Depression Scale; FSS, Fatigue Severity Scale. The data that follow a normal distribution were represented by mean ± standard deviation, and data that do not follow a normal distribution were represented as median and interquartile range.

* Mann-Whitney *U* test.

^#^ Chi-square test.

### Group differences in white matter, gray matter and deep gray matter

**FIGURE 1:**
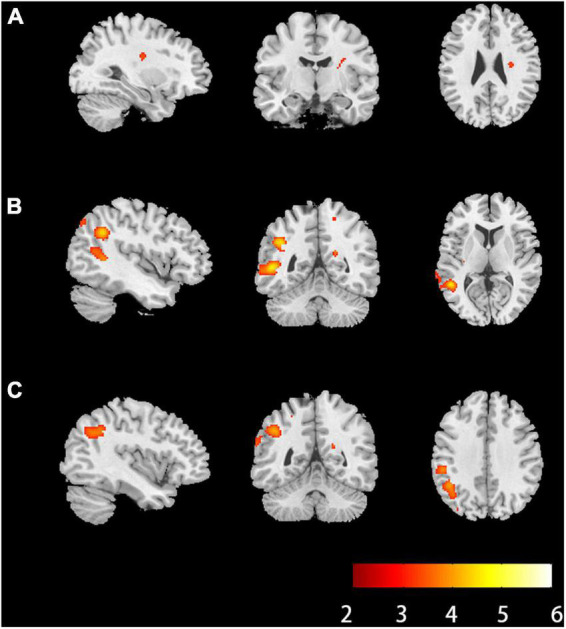
Brain regions of anti-NMDAR encephalitis patients showed significant microstructural changes. **(A)** Brain regions where radial kurtosis (RK) values changed in the white matter (*P* < 0.001, uncorrected, cluster size = 30). **(B)** Brain regions where kurtosis fractional anisotropy values changed in the gray matter (*P* < 0.001, uncorrected, cluster size = 30). **(C)** Brain regions where RK values changed in the gray matter (*P* < 0.001, uncorrected, cluster size = 30). The hot color represents the lower diffusion parameters values in the patients’ group. L, left; R, right; anti-NMDAR, anti-N-methyl-D-aspartate receptor.

Compared with HCs, RK in the right extranuclear which located in white matter significantly decreased in patients with anti-NMDAR encephalitis (*P* < 0.001). Compared with HCs, patients with anti-NMDAR encephalitis displayed significant decrease in KFA in the right precuneus, the right superior parietal gyrus (SPG), the left precuneus, left middle occipital gyrus (MOG) and left superior occipital gyrus (SOG) (*P* < 0.001). Gray matter regions with significant KFA reduction overlapped with those with significant RK reduction in the left middle temporal gyrus (MTG), superior temporal gyrus (STG), supramarginal gyrus (SMG), postcentral gyrus (POCG), inferior parietal but supramarginal gyrus and angular gyrus (IPL) and angular gyrus (ANG) (*P* < 0.001). In the deep gray matter regions, there were no brain regions with diffusion parameters that were significantly different from those of HCs. In addition, compared with HCs, DKI-derived DTI parameters, including FA, MD, AD, RD, and DKI parameters MK and AK were not markedly changed in the brain regions of patients with anti-NMDAR encephalitis. The brain regions of anti-NMDAR encephalitis patients showing significant microstructural changes are shown in Figure 1 and Table 2. These results were obtained by setting the significance level at a cluster level of uncorrected *P* < 0.001, and all results were not significantly different after FDR correction at the statistical significance level set at *P* ≤ 0.05.

**TABLE 2:** Brain regions of anti-NMDAR encephalitis patients showed significant microstructural changes.

Mask	Index	Side	Brian region	Peak MNI coordinates			Peak intensity	Cluster size
						
				X	Y	Z		
WM	RK	R	Extranuclear	24	–14	16	3.8277	69
GM	KFA	L	MTG; STG	–50	–48	6	4.7968	651
		L	SMG	–66	–28	30	3.7275	92
		R	Precuneus	19	–53	21	3.7829	35
		L	ANG; IPL; precuneus; MOG; SOG	–42	50	34	4.4133	570
		L	POCG	–52	–14	44	3.4781	46
		R	SPG	16	–54	60	3.4684	69
	RK	L	STG; MTG	–70	–40	8	3.8475	160
		L	POCG	–62	–6	16	3.5157	47
		L	IPL; SMG	–52	–32	38	4.055	195
		L	IPL; ANG	–44	50	38	4.0567	346

Anti-NMDAR, anti-N-methyl-D-aspartate receptor; RK, radial fractional; KFA, kurtosis fractional anisotropy; WM, white matter; GM, gray matter; MTG, middle temporal gyrus; STG, superior temporal gyrus; SMG, supramarginal gyrus; ANG, angular gyrus; IPL, inferior parietal but supramarginal and angular gyrus; MOG, middle occipital gyrus; SOG, superior occipital gyrus; POCG, postcentral gyrus; SPG, superior parietal gyrus.

### Correlations between diffusion parameters and clinical scale scores

**FIGURE 2:**
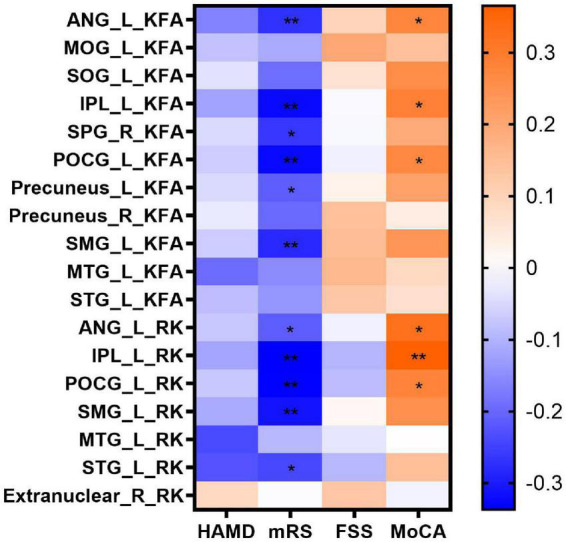
Correlations between clinical scale scores and KFA and RK values in abnormal brain regions in patients with anti-NMDAR encephalitis. Statistical differences are *P < 0.05, **P < 0.01. Anti-NMDAR, anti-N-methyl-D-aspartate receptor; HAMD, Hamilton Depression Scale; mRS, Modified Rankin Scale; FSS, Fatigue Severity Scale; MoCA, Montreal Cognitive Assessment; KFA, kurtosis fractional anisotropy; RK, radial kurtosis; ANG_L, left angular gyrus; MOG_L, left middle occipital gyrus; SOG_L, left superior occipital gyrus; IPL_L, left inferior parietal but supramarginal and angular gyrus; SPG_R, right superior parietal gyrus; POCG_L, left postcentral gyrus; Precuneus_ L, left precuneus; Precuneus_ R, right precuneus; SMG_L, left supramarginal gyrus; MTG_L, left middle temporal gyrus; STG_L, left superior temporal gyrus.

In the correlation analyses, KFA and RK in the left ANG (*r* = 0.277, *P* = 0.037; *r* = 0.323, *P* = 0.014), IPL (*r* = 0.287, *P* = 0.031; *r* = 0.365, *P* = 0.005) and POCG (*r* = 0.270, *P* = 0.042; *r* = 0.280, *P* = 0.035) correlated positively with MoCA scores. KFA and RK in the left ANG (*r* = –0.272, *P* = 0.009; *r* = –0.213, *P* = 0.042), IPL (*r* = –0.326, *P* = 0.002; *r* = –0.337, *P* = 0.001), POCG (*r* = –0.326, *P* = 0.002; *r* = –0.336, *P* = 0.001) and SMG (*r* = –0.283, *P* = 0.007; *r* = –0.313, *P* = 0.003) correlated negatively with mRS scores. In addition, KFA in the left precuneus (*r* = –0.213, *P* = 0.042) and right SPG (*r* = –0.265, *P* = 0.012) as well as RK in the left STG (*r* = –0.242, *P* = 0.021) correlated negatively with mRS scores. No correlation was found between KFA and RK in the abnormal brain regions and HAMD and FSS scores (*P* > 0.05). Correlations between clinical scale scores and KFA and RK values in abnormal brain regions in patients with anti-NMDAR encephalitis are shown in Figure 2 and Table 3.

**TABLE 3:** Correlations between clinical scale scores and KFA and RK values in abnormal brain regions in patients with anti-NMDAR encephalitis.

		HAMD		mRS		FSS		MoCA	
					
		r	*P*	r	*P*	r	*P*	r	*P*
KFA	ANG_L	–0.164	0.222	−0.272**	0.009	0.097	0.475	0.277*	0.037
	MOG_L	–0.081	0.549	–0.113	0.280	0.198	0.140	0.144	0.286
	SOG_L	–0.039	0.773	–0.192	0.067	0.064	0.638	0.258	0.053
	IPL_L	–0.123	0.361	−0.326**	0.002	–0.008	0.953	0.287*	0.031
	SPG_R	–0.049	0.720	−0.265*	0.012	–0.010	0.943	0.193	0.151
	POCG_L	–0.067	0.620	−0.326**	0.002	–0.021	0.879	0.270*	0.042
	Precuneus_L	–0.050	0.711	−0.213*	0.042	0.027	0.843	0.214	0.111
	Precuneus_R	–0.028	0.835	–0.198	0.058	0.144	0.287	0.040	0.767
	SMG_L	–0.067	0.621	−0.283**	0.007	0.150	0.267	0.240	0.072
	MTG_L	–0.195	0.146	–0.152	0.147	0.162	0.230	0.087	0.520
	STG_L	–0.086	0.526	–0.140	0.180	0.127	0.347	0.069	0.608
RK	ANG_L	–0.074	0.587	−0.213*	0.042	–0.019	0.886	0.323*	0.014
	IPL_L	–0.120	0.374	−0.337**	0.001	–0.099	0.465	0.365**	0.005
	POCG_L	–0.074	0.583	−0.336**	0.001	–0.089	0.512	0.280*	0.035
	SMG_L	–0.113	0.401	−0.313**	0.003	0.014	0.915	0.253	0.058
	MTG_L	–0.240	0.072	–0.094	0.370	–0.034	0.804	–0.002	0.988
	STG_L	–0.227	0.089	−0.242*	0.021	–0.094	0.489	0.146	0.279
	Extranuclear_R	0.087	0.519	–0.004	0.969	0.127	0.347	–0.016	0.907

Anti-NMDAR, anti-N-methyl-D-aspartate receptor; HAMD, Hamilton Depression Scale; mRS, modified Rankin Scale; FSS, Fatigue Severity Scale; MoCA, Montreal Cognitive Assessment; KFA, kurtosis fractional anisotropy; RK, radial kurtosis; ANG_L, left angular gyrus; MOG_L, left middle occipital gyrus; SOG_L, left superior occipital gyrus; IPL_L, left inferior parietal but supramarginal and angular gyrus; SPG_R, right superior parietal gyrus; POCG_L, left postcentral gyrus; Precuneus_ L, left precuneus; Precuneus_ R, right precuneus; SMG_L, left supramarginal gyrus; MTG_L, left middle temporal gyrus; STG_L, left superior temporal gyrus.

Statistical differences are *P < 0.05, **P < 0.01.

## Discussion

To the best of our knowledge, this is the first study to investigate the microstructure changes in the whole brain, including white matter, gray matter and deep gray matter in patients with anti-NMDAR encephalitis after the acute stage by applying the DKI method. Our study found that, compared to HCs, patients with anti-NMDAR encephalitis had a distinct pattern of microstructural abnormalities characterized by widespread gray matter damage other than white matter damage, and was correlated with cognitive impairment and the severity of neurofunctional recovery.

### Comparison of diffusion kurtosis imaging and diffusion tensor imaging parameters

In the present study, we found that extensive microstructural changes in patients with anti-NMDAR encephalitis could be quantitatively assessed by DKI parameters. Our results showed that only two kurtosis parameters, RK and KFA changed in patients compared with HCs. Kurtosis values are a series of indices used to describe the complexity of organizational structure. The higher the kurtosis values are, the more restricted and complex the environment is (Das et al., 2017). RK mainly describes the degree to which water molecules are confined in the radial direction and is sensitive to myelin sheath changes (Liang et al., 2021). KFA is an anisotropy parameter measured on the fourth-order tensor based on DKI, similar to FA. FA reflects the difference in the dispersion coefficient in the three axis directions of the ellipsoid and the organizational structure’s directivity, which is positively correlated with the directivity. However, KFA provides more information about anisotropic diffusion dynamics than FA as it is affected by many factors, such as intermolecular distance, cerebrospinal fluid flow, fiber structural integrity, and fiber structural compactness. When KFA is close to zero, the diffusion is close to isotropic. The larger the FKA value is, the closer the organizational structure is (Jensen et al., 2005). It is important to note that changes in diffusion parameters refer to structures such as fiber arrangement and myelin density that can impact the complexity of the environment. In this study, we found that both RK and KFA were decreased in the corresponding brain regions, thus we speculated that the fiber structure arrangement and myelin sheath density of anti-NMDAR encephalitis patients were sparser than those of HCs. However, no abnormalities were detected in brain microstructural changes by DKI-derived DTI parameters when a *P* < 0.001 was set. Therefore, we speculated that DKI parameters were more sensitive than DTI parameters in detecting brain microstructural changes of anti-NMDAR encephalitis patients at the same *P* value threshold. Other studies also supported this speculation. A comparative study in bipolar disorder showed that DKI parameters could detect wider regions and have higher fidelity than DTI parameters at the same P value threshold (Yang et al., 2021). DKI parameters also showed more WM microstructural alternations in patients with poststroke cognitive impairment than DTI parameters revealed (He et al., 2022). Consistent with previous findings, the DKI method greatly exceeded the DTI method for the detection of gray-white matter structures in anti-NMDAR encephalitis patients. This could be attributed to the fact that the DKI algorithm can detect more water molecules that obey a non-Gaussian distribution of diffusion.

### White matter microstructural changes

Our results showed that only the right extranuclear with reduced RK in the white matter regions was found in patients compared with HCs. However, another retrospective study found that patients with anti-NMDAR encephalitis had widespread microstructural alternations with FA decreased in the right MTG, right precuneus, and left middle cerebellar peduncle, and MD increased in the left frontal lobe and MTG (Liang et al., 2020). This might be because the study enrolled patients with a shorter time from clinical onset to MRI scanning (3.1 ± 2.1months) than we did (15.44 ± 9.08months). In addition, the study only included 15 patients and we included a much larger sample with 57 patients. In addition, a case was reported in which a 24-year-old woman with multifocal subcortical white matter lesions in the acute stage showed complete resolution after two months of appropriate immunotherapy (Wang et al., 2015). Therefore, we speculated that the white matter damage in patients with anti-NMDAR encephalitis was not permanent but could be gradually repaired over time. A previous study also indicated that the disease was reversible, immune-mediated neuronal dysfunction rather than irreversible degeneration (Hughes et al., 2010; Dalmau et al., 2011). Although some previous studies using the DTI method reported extensive white matter alterations in anti-NMDAR encephalitis, this appears to conflict with our findings of only RK and KFA changes. However, some of these studies focused on the superficial white matter areas rather than the whole-brain including white-gray matter and deep gray matter areas of recovered and partially recovered patients (Phillips et al., 2018), while others used Tract-Based Spatial Statistic (TBSS) analysis methods that were inconsistent with ours (Finke et al., 2013; Yang et al., 2022). Recently, Wang et al. applied multimodal brain network analysis to show that changes in structural networks were mainly in fiber number and fiber length, and very few significant changes in FA in anti-NMDAR encephalitis (Wang et al., 2021), which is consistent with our findings. While in our research, we didn’t found changes of DTI parameters in white matter. Thus, we speculated that TBSS analysis might be more sensitive than voxel-based analysis in detecting a wide range of white matter microstructural changes of anti-NMDAR encephalitis. As widespread gray matter damage was observed in this study, we speculated that the recovery rate of abnormal white matter microstructure was faster than that of gray matter, or the severity of white matter damage was lower than that of gray matter damage.

### Gray matter microstructural changes and their relationship with cognitive performance

In this study, we found that KFA and RK decreased in the left ANG, IPL and POCG and these changes were correlated with MoCA scores, suggesting that these brain regions play an important role in cognitive impairment in patients with anti-NMDAR encephalitis. Cognitive function is a complex brain activity based on local information processing and effective integration among different brain regions. Therefore, pathological processes can contribute to cognitive dysfunction by directly participating in and disrupting activity in gray matter regions associated with cognitive function (Rocca et al., 2015). The associations between these regions and cognitive damage have been widely reported in previous studies.

The ANG is closely related to a variety of cognitive processes, including spatial cognition, language, number processing, memory retrieval, and attention. From a neuroanatomical perspective, it occupies a central position and acts as a hub of multiple nodes within the default network. It receives input from the sensory cortex and integrates different forms of sensory information (Bonner et al., 2013; Seghier, 2013). ANG injury has been confirmed to produce a variety of cognitive impairments such as aphasia, agraphia and poor arithmetic skills (Price et al., 2018; Sakurai et al., 2021; Liu et al., 2022). These findings supported the function of the ANG in integrating multiple inputs of external information and that changes in the microstructure of the ANG are associated with cognitive impairment.

The IPL is responsible for receiving and integrating information from different modes of physical movement, hearing and vision (Binder et al., 2009). Previous studies have shown that IPL plays an important role in auditory working memory and episodic memory (Mottaghy et al., 2002; Sestieri et al., 2013). In addition, higher pattern similarity in bilateral IPL was found to be associated with better learning performance during the early learning phase (Qu et al., 2021). Recently, a multiparameter study found that neurovascular coupling in the ANG and IPL was decreased and was associated with cognitive deficits (Guo et al., 2022).

The POCG is mainly responsible for integrating the information of various somatosensory stimuli to help individual correctly realize the correct recognition of objects. Some studies have shown that it is involved in cognitive activities. Reduced functional connectivity between the dentate nucleus and the POCG is associated with cognitive impairment in patients with schizophrenia (Xie et al., 2021). A study of patients undergoing cognitive behavioral therapy for mild depression found that increased functional connectivity between the left POCG and the parahippocampal gyrus may be associated with enhanced memory recognition (Du et al., 2016). Previously, a resting state functional MRI found that functional connectivity in the POCG was increased in patients with anti-NMDAR encephalitis after the acute stage. These changes were considered compensation after an injury, or these changes might be caused by the glial cell proliferation at the injury site during recovery (Cai et al., 2020), which contradicts our conclusion that the microstructures of anti-NMDAR encephalitis patients are sparser than those of HCs. Compared with functional sequences, DKI is more able to reflect structural changes, and more patients were included in this study; it is considered that the results in this study may be somewhat more accurate, but the exact changes warrant further pathological study.

### Gray matter microstructural changes and their relationship with disease severity

The mRS scale was used to evaluate the severity of the neurofunctional recovery of patients, which is a comprehensive assessment of neurological functional recovery (Sagnier et al., 2020). This study found that the RK value of the left STG decreased and was associated with mRS scores but not MoCA scores, which may be related to the microstructural damage caused by the high incidence of temporal lobe epilepsy in patients with anti-NMDAR encephalitis (Song et al., 2016; Prager et al., 2019). This finding can also explain the decreased neurovascular coupling of the left STG (Guo et al., 2022). In conclusion, we speculated that the impaired microstructure of the left STG led to its reduced function and low metabolism. This study also found that the left precuneus, left SMG, and right SPG had microstructural changes and were associated with mRS scores. A previous study showed decreased cerebellar blood flow in the precuneus in patients with anti-NMDAR encephalitis (Miao et al., 2020), while this study found that KFA decreased in the left precuneus. Thus, we hypothesized that structural damage to nerve fibers and other structures of precuneus led to a decrease in metabolism. The SMG participates in action planning, and its impairment can lead to mobility impairments (Króliczak et al., 2016). Therefore, reducing RK and KFA in the SMG may be associated with impaired motor function in patients with anti-NMDAR encephalitis.

### Gray matter microstructural changes and the relationship with depression and fatigue

This study did not find any correlation among KFA and RK in the abnormal brain regions, HAMD scores and FSS scores, which means that microstructural damage may not be associated with increased depression or fatigue. Another resting-state functional MRI study also did not find correlations between abnormal functional connectivity and depression (Cai et al., 2020). However, we did not find other studies demonstrating a correlation between fatigue and structural or functional abnormalities in patients with anti-NMDAR encephalitis. It is clear that many patients are chronically depressed or prone to fatigue after the acute stage, but no correlation has been found in functional or structural sequences. It is possible that these changes are more subtle and will require more sensitive techniques to detect them in the future.

### Laterality of patients’ microstructural changes

It is interesting that the brain regions with microstructural changes are primarily located in the left hemisphere. Thus, we speculated that the effects of anti-NMDAR encephalitis on the brain are asymmetrical. This might be because NMDARs are unevenly distributed on both sides of the brain (Ferreira et al., 2020). For a right-handed person, the left hemisphere is their dominant hemisphere, responsible for language production and comprehension, motor processing, and logical interpretation (Rentería, 2012). As all of our patients are right-handed, the impact of anti-NMDAR encephalitis on these functions mentioned above is significant. Studies have already found that changes in lateralization of the cortex are a prominent marker of several neurological disorders, such as schizophrenia, Alzheimer’s disease, and depression (Sheng et al., 2013; Liu et al., 2018; Yang et al., 2022), and may also be a well-known marker of anti-NMDAR encephalitis.

### Limitations

There were several limitations to this study. First, to detect larger activated areas of gray matter and white matter, we used a Gaussian kernel of 6 × 6 × 6 mm FWHW. However, applying a larger kernel has the disadvantage of reducing its ability to detect smaller regions. In our study, the diffusion parameters in deep gray matter regions were not significantly different patients and HCs. This result might be due to the large smoothing kernel applied to the deep gray matter regions resulting in detectability reduced. To date, little attention has been specifically paid to the deep gray matter in patients with anti-NMDAR encephalitis. Applying a smaller Gaussian kernel and combining other techniques to further explore the changes of deep gray matter in patients with anti-NMDAR encephalitis are needed in the future. Second, cognitive function can be divided into different cognitive domains, with different pathological substrates that contribute to the disease. In this study, we only used MoCA scores to assess the overall cognitive function of the subjects, a more refined cognitive scale are needed in the future. Third, the mRS scale was used to comprehensively evaluate the severity of the neurofunctional recovery of patients. This study speculated that, in addition to cognitive function impairment, patients with anti-NMDAR encephalitis still had impaired motor and sensory functions after the acute stage. Future adoption of specialized scales for assessing motor dysfunction, such as the Motor Assessment Scale, will be considered. Nevertheless, this study did not use the scale, especially for motor and sensory function evaluation. Finally, patients with anti-NMDAR encephalitis included in this study had different disease durations. They are in different stages after acute phase, which may have resulted in different degrees of damage and microstructures repair.

## Conclusion

In conclusion, by means of the DKI method, our study demonstrated that the microstructural changes in gray matter were much more extensive than those in white matter in patients with anti-NMDAR encephalitis after the acute stage. The brain damage reflected by DKI parameters, which have higher sensitivity than DTI parameters, was correlated with the cognitive impairment and severity of the neurofunctional recovery, which may help to assess the severity and progression of this disease.

## Data availability statement

The original contributions presented in this study are included in the article/supplementary material, further inquiries can be directed to the corresponding authors.

## Ethics statement

The studies involving human participants were reviewed and approved by the Institutional Review Board of The First Affiliated Hospital of Chongqing Medical University, Chongqing, China. The patients/participants provided their written informed consent to participate in this study.

## Author contributions

JF and JL offered the study design. SD and JL collected all the data. YX and SD offered the data processing support. HL and YX contributed to writing the first draft of the manuscript. CZ, TL, and YL provided the guidance and critical reviews. All authors contributed to the article and approved the submitted version.

## Footnotes

1
http://www.mricro.com/

2
http://www.fmrib.ox.ac.uk/fsl/

3
http://www.nitrc.org/projects/dke/

4
http://www.fil.ion.ucl.ac.uk/spm/
